# Interrelationships of the Intestinal Microbiome, Trimethylamine N-Oxide and Lipopolysaccharide-Binding Protein with Crohn’s Disease Activity

**DOI:** 10.3390/pathogens14010005

**Published:** 2024-12-27

**Authors:** Yelena Laryushina, Nadezhda Samoilova-Bedych, Lyudmila Turgunova, Alexandr Marchenko, Yermek Turgunov, Samat Kozhakhmetov, Maxat Suieubayev, Nurislam Mukhanbetzhanov, Nadezhda Kabdulina

**Affiliations:** 1 Department of Internal Diseases, Karaganda Medical University, Karaganda 100000, Kazakhstan; 2 National Laboratory Astana, Nazarbayev University, Astana 010000, Kazakhstan; 3 Gastroenterology Department, Regional Clinical Hospital, Karaganda 100000, Kazakhstan

**Keywords:** Crohn’s disease, CD, IBD, trimethylamine N-oxide, lipopolysaccharide-binding protein, TMAO, LPS, gut microbiome, intestinal microbiome

## Abstract

Crohn’s disease (CD) is a multifactorial inflammatory bowel disease whose pathogenetic mechanisms are a field of ongoing study. Changes in the intestinal microbiome in CD may influence metabolite production and reflect the disease’s severity. We investigate the relationship between trimethylamine N-oxide (TMAO) and lipopolysaccharide-binding protein (LPS) levels and changes in the gut microbiome in patients with CD of various degrees of activity. Methods: In total, 29 CD patients and 15 healthy individuals were investigated for their levels of TMAO by HPLC-MS, and LPS protein by ELISA and metagenomic 16 s-sequencing of feces was performed. Results: We found significant differences in TMAO levels in patients in the remission/mild and moderate/severe groups compared to the control group (*p* = 0.02 and *p* = 0.014), changes in alpha diversity with the Shannon index (*p* = 0. 0151 and *p* = 0.0018) and in beta diversity (ANOSIM *p* = 0.009 and PERMANOVA *p* = 0.005) in both groups compared to controls. Strongly positive correlations in TMAO levels and mixed correlations of LPS with alpha diversity metrics were found, as well as significant correlations with microbiota species. Conclusions: Changes in the level of metabolites may reflect specific disturbances in the composition of the intestinal microbiome at different degrees of severity of CD.

## 1. Introduction

Crohn’s disease (CD) is a chronic inflammatory bowel disease (IBD), which is characterized by the involvement of various parts of the gastrointestinal tract with progressive inflammation of all layers of the intestinal wall leading to the formation of ulcers, stenoses, and abscesses. Despite the active study of the mechanisms of disease development, the etiology and pathogenesis of CD remain completely unclear. In recent years, more and more attention has been paid to the development and progression of the disease and the role of the intestinal microbiome, as well as its metabolites such as trimethylamine N-oxide (TMAO) and lipopolysaccharide-binding protein (LPS protein).

The microbiome is a complex community of microorganisms including bacteria, viruses, fungi, and archaea that perform key functions in digestion, metabolism, and regulation of the body’s immune system. Changes in the composition of these microbial communities may act as a key factor in the pathogenesis of CD [1] by decreasing the abundance of anti-inflammatory agents [2] and increasing bacteria with pathogenic properties [3], having a significant impact on the development and progression of the disease [4].

Trimethylamine N-oxide (TMAO) is a metabolite formed from the microbial processing of choline, carnitine, and other compounds found in food. Recent studies show that elevated levels of TMAO are associated with an increased risk of cardiovascular disease and inflammation. This metabolite is related to endothelial and vascular dysfunction in patients with CD [5]. Wilson et al. considered TMAO as a potential biomarker of IBD and CD and found decreased TMAO levels in affected individuals compared to controls. Still, they found no difference in concentration in clinically active and inactive forms of CD [6]. LPS protein plays a key role in recognizing and binding lipopolysaccharides (LPS), components of the outer membrane of Gram-negative bacteria. Increased blood levels of LPS are associated with increased intestinal barrier permeability, which may contribute to systemic inflammation. It has been demonstrated that LPS protein levels may correlate with CD activity, and its elevated concentrations are associated with an increased risk of relapse in patients [7].

The influence of microbiome changes on TMAO and LPS protein levels is considered one of the possible reasons for the increased inflammatory response in patients with active CD. Thus, a decrease in the abundance of LPS-degrading bacteria such as *Bacteroides* may contribute to an increase in systemic LPS concentration and activation of the immune response [8]. At the same time, an increased abundance of TMAO-producing bacteria, such as *Clostridia*, may increase inflammation through mechanisms related to NLRP3-inflammasome activation and the production of proinflammatory cytokines [9]. The interaction between these metabolites and the microbiome emphasizes the need for a comprehensive approach to the study of disease pathogenesis that considers not only inflammatory processes but also changes in the metabolic profile and microbiota of patients. Studies on the relationship between the composition of intestinal microbiota and TMAO concentrations in patients with different degrees of CD activity are not currently presented.

This study aims to investigate the relationship between TMAO and LPS protein levels and changes in the intestinal microbiome composition in patients with CD of various degrees of activity.

## 2. Materials and Methods

In this study participated 29 patients with CD and 15 healthy control subjects. Recruitment of participants was undertaken at the Non-commercial joint-stock company (NCJSC) “Karaganda Medical University” medical university clinic. Participants had no restrictions on gender, race, or ethnicity. Individuals under the age of 18 years, those with severe psychiatric and neurologic diseases and chronic pathologies (cardiovascular, endocrinological), and pregnant women were not included in the study. All study participants had lived in the Karaganda region for at least the last year. Each participant signed an informed consent form approved by the Local Bioethics Committee (Minutes 9 of the meeting of the Local Bioethics Committee of 29 March 2022, Minutes 1 of the meeting of the Local Bioethics Committee of 22 September 2022). During clinical examination, the previous intake of antibiotics, probiotics, and NSAIDs was clarified in each participant. If patients had taken drugs from these groups in the previous 3 months, they were excluded from the study. The representatives of the experimental group had a confirmed diagnosis of CD according to clinical, laboratory, endoscopic, and histologic criteria of the disease. To exclude the influence of nutritional status on the results of the study, all participants completed the GIVES-21 nutrition screener questionnaire [10]. The questionnaire included 9 questions, analyzing the amount of food eaten in the last 7 days by food category.

Biomaterial was obtained from each participant: venous blood and feces. The fecal sample collection was performed by participants at home with prior instruction. The most important condition was to deliver feces no later than 60 min after the act of defecation for subsequent freezing at ultra-low temperature (−80 °C) of one of the two collected fecal containers. Venous blood was obtained from patients by venipuncture in the procedure room by a nurse practitioner. Blood tests were performed on Sysmex XN-2000 (Sysmex Corporation, Hyogo Kobe Japan) and Beckman Coulter (Beckman Coulter, Brea, CA, USA) analytical systems, and fecal calprotectin was analyzed with the ELISA method using a EliA Calprotectin 2 test system by Phadia GmBH (Thermo Fisher Scientific, Freiburg, Germany).

Determination of TMAO and LPS protein was carried out in the Research Laboratory of NCJSC “KMU”. HPLC-MS/MS determined the TMAO level on Agilent 1260 Infinity (Agilent Technologies, Santa Clara, CA, USA) and G6130A quadrupole LC/MS (Agilent Technologies, Santa Clara, CA, USA) systems. Venous blood in an EDTA tube was immediately centrifuged at 3000 rpm for 15 min. Further, the obtained plasma was frozen and stored in freezers at −80 °C. The following solutions were used for the assay: trimethylamine N-oxide 95% (Sigma-Aldrich, St. Louis, MO, USA), formic acid 95%, (Sigma-Aldrich, St. Louis, MO, USA), acetonitrile 99.9% (Sigma-Aldrich, St. Louis, MO, USA), and high purity water (18.2 mg/L) obtained from the Milli-Q system (Millipore, Burlington, MA, USA). For the assay, 600 μL of acetonitrile was added to 100 μL of thawed plasma followed by centrifugation at 20,000× *g* for 10 min at 4 °C. Then, 100 μL of water was added to 100 μL of the resulting supernatant, and the resulting mixture was injected into an HPLC-MS/MS system with a sample volume of 10 μL. Analysis was performed using an Agilent 1260 Infinity HPLC system (Agilent Technologies, Santa Clara, CA, USA), and a G6130A quadrupole LC/MS (Agilent Technologies, Santa Clara, CA, USA) ZORBAX Eclipse XDB system (Agilent Technologies, Santa Clara, CA, USA), 80 °C 18, 2.1 × 75 mm, 3.5 μm particle size, and Zorbax Eclipse XDB C-18, 12.5 × 4.6 mm, 3.5 μm guard column were used for separation. Isocratic separation was performed using eluents A (0.125% formic acid solution in acetonitrile at a ratio of 1:1) and B (0.125% formic acid solution in water at a ratio of 1:1) at 30 °C at a flow rate of 0.250 mL/min. Calculation of the TMAO level was performed after measuring the area of the obtained graph.

LPS protein determination was performed by enzyme immunoassay on a Biorad EVOLIS robotic station (Hercules, CA, USA) using an ELISA Kit (Cloud-Clone Corp., Export Processing Zone, Wuhan, Hubei, China) for lipopolysaccharide-binding protein. After venous blood collection (clotting activator tube) and sample delivery, whole blood was centrifuged for 20 min at 1000 rpm. Aliquoted samples of the resulting serum were frozen and stored at −80 °C in ultra-low temperature freezers until analysis. Samples were thawed at room temperature before analysis. After the preparation of sample reagents and standards, 100 μL of standard/sample was added to each well. Incubation at 37 °C was performed for 60 min. Next, aspiration was performed and 100 µL of prepared detection reagent A was added followed by incubation for 60 min at 37 °C. This was followed by aspiration and washing 3 times. Subsequently, 100 μL of the prepared detection reagent B was added and incubated for 30 min at 37 °C, aspirated, and washed 5 times. After that, 90 μL of substrate solution was added and incubated for 10–20 min at 37 °C, 50 μL of stop solution was added, and an immediate reading was performed at 450 nm.

Patients with CD were scheduled for colonoscopy within 7–10 days of their first visit. The procedure was performed by an experienced endoscopist with more than 5 years of experience with patients with IBD. The examination was performed using an Olympus CF-H170L video colonoscopy stand (serial number 282,303–Olympus, Shinjuku, Tokyo, Japan).

The intestinal microbiome composition was analyzed in the National Laboratory Astana, Nazarbayev University. After delivery to the laboratory, fecal samples from patients with ulcerative colitis and control volunteers were placed in DNA/RNA Shield-Fecal Collection Tube kits (Cat. No.: R1101, Zymo Research Corporation, Tustin, Hercules, CA, USA) with a total volume of 10 mL according to the manufacturer’s instructions. ZymoBiomics DNA Microprep (Cat. No.: D4300, Zymo Research Corporation) was used for microbial DNA purification. For standardized DNA extraction, strictly 0.2 mL of the sample was extracted. Total DNA extraction was checked in 1% agarose gel, and DNA concentration was measured using a Nabi UV/Vis Nano Spectrophotometer (MicroDigital, Gyeonggi-do, Seongnam, Republic of Korea). Metagenomic sequencing of 16 S Amplicon was performed in the laboratory on the Illumina NovaSeq6000 platform according to the metagenomic sequencing protocol. Primary bioinformatics analysis was performed using the less operational taxonomic single script 2 (LotuS2).

Primary bioinformatics analysis was performed using the less operational taxonomic single script 2 (LotuS2). The initial microbiological data analysis was performed in a Python (3.8.18 version) environment employing specialized statistical and bioinformatic tools. Alpha diversity was assessed using the Shannon, Simpson, Observed OTUs, and Pielou’s evenness indices. Comparisons of alpha diversity among groups were conducted using the non-parametric Kruskal–Wallis test, followed by Tukey’s post hoc test for pairwise differences. Additionally, one-way ANOVA with Tukey’s test was applied for pairwise comparisons. Beta diversity was determined based on Bray–Curtis dissimilarity and evaluated using PERMANOVA (R = 2.355, *p* = 0.005) and ANOSIM (R = 0.137, *p* = 0.009), with visualization achieved through Principal Coordinates Analysis (PCoA). Correlations between microbial community parameters (alpha diversity and relative taxa abundances) and metabolite concentrations (TMAO, LPS) were explored using Spearman’s non-parametric correlation. The LEfSe method was employed to identify taxa discriminating among sample groups.

Data entry and primary statistical processing were performed using the MS Excel program from the Microsoft Office suite of programs (version 2306 (Build 16529.20154)). Further, the patient’s data were analyzed in the IBM SPSS Statistics 22 program. The Kolmogorov–Smirnov test (K-S test) was used to assess the normality of data distribution. Data with normal distribution were described by the mean with standard deviation if the data were not normally distributed by the median and interquartile range. Data were compared using parametric and non-parametric criteria based on the normality of distribution. The chi-square test was used to analyze qualitative data, and the Mann–Whitney U-test and Kruskal–Wallis test were used to analyze quantitative data. Spearman’s correlation analysis test was used to calculate mutual relationships. A *p*-value of less than 0.05 (significance level α = 0.05) was considered statistically significant. Python (3.8.18 version) was used for data analysis. The primary libraries utilized included pandas (version 2.2.3) for data manipulation and processing, scipy (version 1.14.1) for statistical testing, scikit-learn (version 1.5.2) for machine learning methods, and numpy (version 2.1.1) for numerical calculations. Data visualization was performed using the matplotlib library (version 3.9.2).

Data filtering was conducted using two approaches. The first approach, “Filtering of taxa based on the threshold variability of relative abundance with a threshold of 0.01”, was used to exclude taxa with low variability in abundance across samples. The second approach, “Filtering of taxa based on the threshold value of relative abundance with a threshold of 0.00001”, removed taxa whose presence was below a specified minimum threshold.

Statistical analysis included the Kruskal–Wallis test for group comparisons and Tukeyhsd MultiComparison for multiple comparisons. To assess differences in microbiome structure between groups, the PERMANOVA and ANOSIM methods were applied.

## 3. Results

A total of 54 patients participated in the study, of whom 29 patients had a confirmed diagnosis of Crohn’s disease and 15 were control volunteers (Table 1). The mean age of the patients was 43 years in the main group and 41 years in the control group. Both groups were predominantly female. The groups had no differences in the sex and age composition (*p* = 0.644 and *p* = 0.651, respectively).

Most patients had an inflammatory course, and a significantly smaller number of patients had a stricturing and penetrating course. The assessment of disease activity was performed according to the CDAI scale using a calculator to calculate the sum of scores on clinical and laboratory indications and the presence of complications [11]. Further, according to the sum of scores, patients were divided into two groups: CDAI-I, patients with a sum score of less than 220, and CDAI-II, those with 220 points or more. As a result, the CDAI-I group included 18 patients with CD in remission and minimal activity, and the CDAI-II group included 11 patients with moderate and severe degrees of clinical and laboratory activity of the disease.

When analyzing foods consumed in the last week, according to the GIVES-21 questionnaire, patients with CD had differences in the consumption of the number of fruits and vegetables and alcohol-containing beverages compared to the control group. The amount of consumption of food categories (red meat and its products, fish, milk and dairy products, eggs, whole grains) rich in natural choline, carnitine and phosphatidylcholine did not differ between the main and control groups (Table 2).

Laboratory parameters were evaluated in each group depending on the disease activity and compared with the control group. Significant differences were obtained in indicators such as albumin level and fecal calprotectin. Also, the level of CRP in patients with marked activity differed from healthy individuals. The indicators are presented in Table 3.

**Figure 1:**
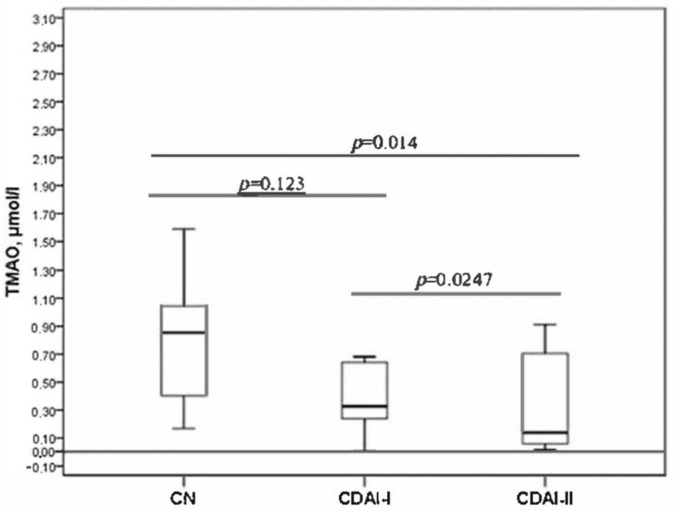
TMAO level in CDAI-I, CDAI-II and Control groups (CN).

The serum TMAO level decreased with increasing disease activity (Figure 1). The level of TMAO was statistically significantly different between the CDAI-I and CDAI-II groups. Significant differences were also revealed when comparing the TMAO level in the CDAI-II group with the control group. The CDAI-I group did not statistically differ from healthy individuals in terms of TMAO level.

The TMAO levels in both groups divided by severity showed significant differences compared to the controls. Also, TMAO differed between the groups. It was higher in patients in remission and with low activity and differed significantly from patients with moderate and severe disease (Figure 1).

**Figure 2:**
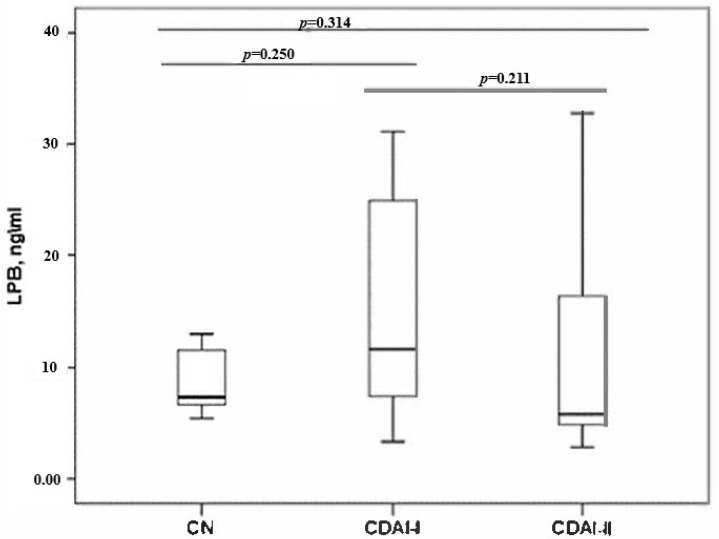
LPS level in CDAI-I, CDAI-II, and control groups (CN).

When assessing the LPS protein, no statistically significant differences were found between the groups by disease activity and with the control group (Figure 2).

**Figure 3:**
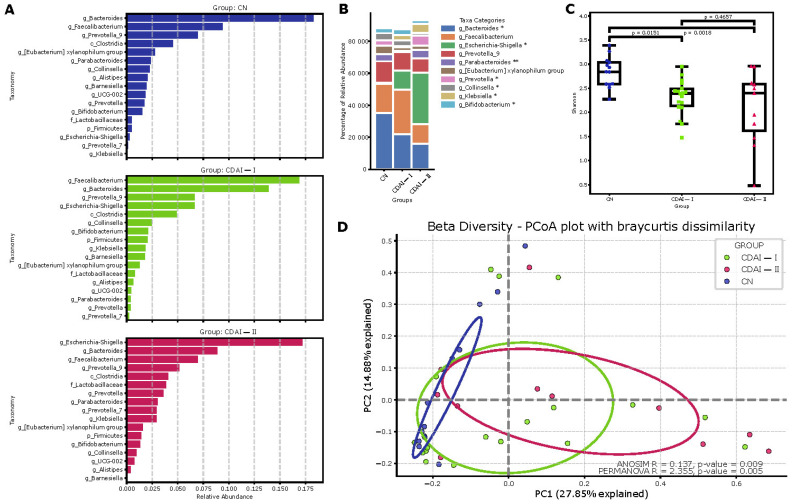
(**A**) Main drivers of Dirichlet multinomial model-based Crohn’s disease communities (CN: control group; CDAI-I: remission or mildly active disease, <220; CDAI-II: severe disease, >220). (**B**) Top 10 taxa. (**C**) Alpha diversity (Shannon index). (**D**)—Beta diversity (Bray–Curtis dissimilarity). * *p* < 0.05, ** *p* < 0.001.

Comprehensive analysis of the gut microbiome in CD patients and healthy individuals demonstrates differences in microbial community composition and diversity (Figure 3). Analysis of the main drivers in the Dirichlet multinomial model (Figure 3A) details the relative abundance of the main taxa in each group, in descending order relative to abundance in all samples. *Bacteroides* and *Faecalibacterium* dominate the control group (CN), whereas the CDAI-I and CDAI-II groups show an increase in the proportion of *Escherichia-Shigella* and a decrease in the proportion of *Faecalibacterium*. These changes were particularly pronounced in the CDAI-II group, consistent with the concept of progressive dysbiosis with increasing CD activity.

Principal coordinates analysis (PCoA) based on the Bray–Curtis distance (β-diversity) confirmed significant compositional differences between the groups of controls (CN), CD patients in remission or with low disease activity (CDAI-I, <220) and patients with moderate to severe CD (CDAI-II, >220). Statistically significant differences between groups were confirmed by the ANOSIM (R = 0.137, *p* = 0.009) and PERMANOVA (R = 2.355, *p* = 0.005) results, indicating significant differences in microbiome composition between the study groups (Figure 3D).

Determination of the relative abundance of major taxa in each group (Figure 3B) showed significant differences in the representation of some genera, *Bacteroides*, *Escherichia-Shigella*, *Parabacteroides*, *Prevotella* and *Collinsella*. These data suggest a dysbiosis of the microbiome in CD, particularly pronounced in the severe course of the disease.

Evaluation of microbiome alpha diversity based on the Shannon index (Figure 3C) revealed a statistically significant decrease in diversity in the CDAI I and CDAI II groups compared to the control group (*p* = 0.0151 and *p* = 0.0018, respectively). This indicates a decrease in microbial diversity in CD, which becomes more pronounced with increasing disease severity.

**Figure 4:**
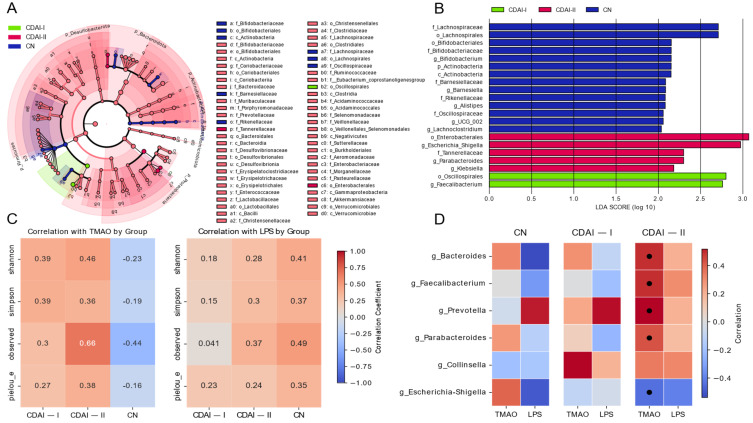
Analysis of microbiome composition, biomarkers, and disease activity. (**A**) Linear discriminant analysis (LDA) effect size (LEfSe) showing differentially abundant taxa between CDAI-I (mild-to-moderate Crohn’s disease), CDAI-II (severe Crohn’s disease), and CN (healthy controls) groups. The length of each bar represents the log10 transformed LDA score. (**B**) Heatmap depicting correlations between key bacterial genera and biomarkers (TMAO and LPS) across different disease activity groups. Red indicates positive correlation; blue indicates negative correlation. (**C**) Alpha diversity metrics (Shannon index, Simpson index, observed species, and Pielou’s evenness) correlated with TMAO levels across disease activity groups. (**D**) Alpha diversity metrics correlated with LPS levels across disease activity groups. In panels (**C**,**D**), the color intensity and size of circles represent the correlation strength, with red indicating positive and blue indicating negative correlations. CDAI: Crohn’s Disease Activity Index; TMAO: trimethylamine N-oxide; LPS: lipopolysaccharide binding protein.

The effect size (LEfSe) linear discriminant analysis (LDA) analysis (Figure 4A) reveals significant taxonomic differences between healthy individuals (CN), patients in remission and with mild CD activity (CDAI-I) and patients with moderate to severe CD (CDAI-II). Notably, the genus *Faecalibacterium* shows the highest LDA in the CDAI-I group, consistent with its known anti-inflammatory properties. In contrast, the CDAI-II group is characterized by a higher prevalence of potentially proinflammatory taxa including *Klebsiella* and *Escherichia-Shigella*. These results are consistent with the concept of progressive dysbiosis with increased CD activity, as observed in the Dirichlet multinomial model analysis (Figure 3A).

Correlations of alpha diversity metrics with TMAO and LPS levels (Figure 4C) provide further insight into the relationship between microbial diversity and these metrics. In the CDAI-II group, TMAO shows a strong positive correlation with all alpha diversity metrics, especially with the observed number of species. This observation is intriguing given the lower levels of TMAO observed in CD patients compared to healthy control subjects. It is hypothesized that reduced TMAO levels in patients with moderate to severe disease activity reflect dysbiosis and altered numbers of certain members of the microbiome, potentially indicating a complex relationship between TMAO, microbial diversity and disease activity.

Correlations of LPS with alpha diversity scores (Figure 4C) show a different pattern, with mostly positive correlations in the CN group and weak correlations in the CD groups. This difference in correlation patterns between TMAO and LPS emphasizes the different roles these indices may play in CD pathophysiology.

The heatmap (Figure 4D) illustrates the correlations between key bacterial genera and the TMAO and LPS biomarkers in different disease activity groups. Members of *Bacteroides*, *Prevotella*, and *Parabacteroides* show a weakly positive correlation with TMAO in the CN group but a strong positive correlation in the CDAI-II group. Interestingly, *Faecalibacterium* has a strong positive correlation in patients in the CDAI-II group. These shifts may reflect changes in microbial metabolism or host–microbiome interactions with disease progression. The negative correlation between *Escherichia-Shigella* and TMAO and LPS in the groups, increasing with increasing disease activity, is consistent with a potential role of these bacteria in promoting inflammation. LPS showed less pronounced correlations, with weakly positive relationships in the group with CDAI-II.

## 4. Discussion

The microbial balance of the gut microbiome plays a huge role in maintaining a healthy gut environment. Host physiology can be altered at the cellular level through microbiome-induced cell signaling, proliferation, and neurotransmitter biosynthesis, resulting in mucosal and systemic changes and thereby affecting homeostasis, barrier function, innate and adaptive immune responses, and metabolism. Changes in the composition of microbial communities lead to disturbances in the biosynthesis of metabolites and have a significant impact on the development and course of CD. Worldwide evidence favors that one of the main factors influencing variations in microbiome composition is geographical location [12,13]. Clooney et al. showed in their intercontinental study that geographic location has the greatest influence on the composition of the intestinal microbiome in patients with and without a diagnosis of CD [14].

To our knowledge, this is the first study to collectively characterize the composition of the gut microbiome in CD patients and the levels of TMAO and LPS protein metabolites and to evaluate their relationship with disease activity.

Our results demonstrate significant differences in gut microbiome composition and diversity between healthy individuals and CD patients, as well as between patients with different disease activity levels. In CD patients, we observed an increase in *Escherichia-Shigella* proportion (opportunistic flora with proinflammatory activity) and a decrease in anti-inflammatory *Faecalibacterium* and *Bacteroides*. These findings align with previous studies reporting elevated levels of potentially proinflammatory bacteria, particularly *Escherichia/Shigella*, in CD patients compared to healthy controls [15,16,17]. Conversely, CD patients show a marked decrease in anti-inflammatory *Faecalibacterium* [16,18]. This dysbiosis extends beyond CD patients; healthy relatives also exhibit altered microbiota composition compared to healthy controls [19]. The decrease in the abundance of anti-inflammatory bacteria (*Faecalibacterium prausnitzii*) and increase in pathogens (*Escherichia coli*) correlate with higher disease activity and symptom severity [2]. The group with moderate and high CDAI activity demonstrated higher prevalence of potentially proinflammatory taxa (*Klebsiella* and *Escherichia-Shigella*) and decreased *Faecalibacterium*, consistent with progressive dysbiosis in increasing CD activity. Morgan et al. showed that dysbiosis in CD is most often characterized by changes in Firmicutes and Proteobacteria types [20], with these variations occurring most frequently in the early stages of the disease. At the same time, during disease progression, pathogenic genera such as *Escherichia/Shigella* and *Enterococcus* dominate, which is associated with increased inflammatory processes, the progression of inflammatory processes and worsening clinical condition [3]. A study by Santana p. et al. demonstrates that high levels of bacteria of the *Escherichia* and *Shigella* genera were associated with more frequent and severe exacerbations of the disease [21]. These findings could serve as a basis for evaluating treatment response, as previous studies showed better clinical remission in patients with increased *Lactobacillus* and *Bifidobacterium* levels [17,22].

Changes in the composition of the microbiome also affect the production of metabolites. Studies on the concentration of TMAO in CD are very limited. The study by Wilson et al. [6] found no differences in TMAO concentrations between patients with active and inactive CD stratified based on clinical parameters. We obtained evidence that TMAO levels not only decrease in CD compared to healthy individuals but also found that they decrease with increasing CDAI activity index. Perhaps these differences in results are to some extent due to the different approaches in determining the degree of CD activity.

Notably, we demonstrated for the first time TMAO correlations with gut microbiome representatives in CD patients. We observed a negative correlation between *Escherichia-Shigella* and TMAO across disease activity groups, more pronounced in moderate and severe disease. This contrasts with other conditions—in heart failure patients, *Escherichia/Shigella* abundance is positively correlated with TMAO levels [23], and similarly in frail elderly individuals [24]. This inverse relationship in CD patients suggests that decreased TMAO may reflect disease-specific changes in gut microbiome composition, particularly the predominance of proinflammatory *Escherichia-Shigella* in the context of inflammatory bowel disease.

Simultaneously, positive correlations between TMAO and anti-inflammatory Bacteroides and Faecalibacterium suggest that as these bacteria decrease, metabolite levels decrease with increasing CD activity. Notably, *Actinobacteria-Colinsella* showed a strong positive TMAO correlation in the CDAI-I group and a weaker but positive correlation in CDAI-II patients. While previous studies identified potential TMA synthesis pathways in various bacteria [25,26,27], these were based on genomic databases without disease context. The positive relationships we found between *Prevotella*, *Parabacteroides*, and TMAO in the CDAI-II group warrant further investigation. Thus, our study provides a basis for speculation that other microorganisms may be involved in TMAO metabolism, and the influence of other mechanisms cannot be excluded.

Regarding LPS, despite its known role in inflammatory responses through TLR4 receptor interaction [28,29], we found no increased LPS protein levels in CD patients compared to healthy individuals, nor with increasing disease activity. This aligns with previous studies showing stable LPS levels in IBD patients [30]. Although our cohort included moderate to severe CDAI patients, inflammatory markers (leukocytes, ESR, CRP) showed no significant group differences, suggesting a possible absence of endotoxemia. However, we observed *Escherichia-Shigella* and LPS correlation in the CDAI-II group, supporting their role in inflammation through LPS production [28].

Several limitations should be acknowledged. First, our sample size (29 CD patients, 15 controls), while showing sufficient statistical power (achieved power 1.00), may limit the result’s generalizability. Second, the cross-sectional design prevents the assessment of temporal changes in microbiome composition and metabolite levels. Third, while we controlled for several confounders through study design (dietary patterns via GIVES-21 questionnaire, medication use, geographic location), some potential confounding factors require consideration.

Despite these limitations, our study provides valuable insights into relationships between gut microbiota composition, metabolite levels, and CD activity. The differential abundance of certain taxa, their correlations with TMAO and LPS, and complex relationships between these markers and microbial diversity emphasize CD pathogenesis’s multifaceted nature. These findings are consistent with recent studies emphasizing the role of altered gut microbial composition in CD and suggest potential mechanisms by which changes in the gut microbiome may contribute to disease activity and disease progression. Our findings provide a fundamental basis for developing novel therapeutic interventions and approaches to diagnose and predict CD course.

## Figures and Tables

**Table 1:** Clinical and demographic characteristics of Crohn’s disease patients and healthy controls.

Characteristic	Crohn’s Disease (*n* = 29)	Healthy Controls (*n* = 15)	*p*-Value
Age, years (mean ± SD)	43.03 ± 12.65	41.33 ± 9.69	0.644
Sex, n (%)			0.651
- Male	11 (38%)	4 (27%)	
- Female	18 (62%)	11 (73%)	
Disease phenotype, n (%)		N/A	
- B1 (inflammatory)	22 (75.9%)		
- B2 (stricturing)	5 (17.2%)		
- B3 (penetrating)	2 (6.9%)		
CDAI, n (%)		N/A	
- Remission (<150)	5 (17.2%)		
- Mild activity (150–220)	13 (44.8%)		
- Moderate activity (220–450)	8 (27.6%)		
- Severe activity (>450)	3 (10.3%)		

SD—standard deviation, CDAI—Crohn’s Disease Activity Index, N/A—not applicable.

**Table 2:** Assessment of nutrition of patients with CD and Healthy control.

Food Products	CD, Median	Control, Median	*p*-Value
Meat, red meat and meat-containing products, gr/week	1162	1232	0.214
Fatty fish, gr/week	144	145.6	1.012
Dairy products, gr/week	598	653	0.674
Eggs (1 egg = 64 gr.), gr/week	322	384	0.714
Cereals, bread, and bakery products, gr/week	506	546.2	0.269
Fruits, gr/week	322	688.3	0.032
Vegetables, gr/week	396	539	0.012
Alcohol (1 portion: 250 mL of beer or 100 mL of wine or 30 mL of spirits), portions/week	1	3	<0.001

CD—Crohn’s Disease

**Table 3:** Laboratory characteristics of the CDAI-I, CDAI-II and healthy control (CN) groups.

Blood Parameter	CDAI-I, Median [IQR]	CDAI-II, Median [IQR]	CN, Median [IQR]	*p*_1_-Value	*p*_2_-Value
Hemoglobin, g/L	125 [113–145]	119 [107–145]	135 [125–145]	0.781	0.548
Hematocrit, %	37.5 [31–41.5]	34 [29.5–39]	41 [33–48]	0.651	0.116
WBC, 109/L	6.9 [5.1–8.5]	6.8 [5.8–9.3]	6.1 [4.9–7.25]	0.426	0.527
ESR, mm/h	11 [6–27]	20.5 [12.5–38.7]	8 [2—11.2]	0.001	0.006
CRP, g/L	1.7 [1.0–7.7]	5.9 [0.87–20.5]	1 [0.5–2.2]	0.845	<0.001
Fecal calprotectin	238.66 [50.12–909.53]	828 [185.6–2489]	36 [13–42]	<0.001	<0.001
Albumin, g/L	44.1 [41–48.5]	37.8 [30.05–44.27]	46 [43.8–49.3]	<0.001	<0.001

*p*_1_—*p*-level between CDAI-I and CN; *p*_2_—*p*-level between CDAI-II and CN.

## Data Availability

Deidentified information data presented in this manuscript will be made available 6 months after publication on reasonable request by email to the corresponding author for research purposes.
